# Season of Gratitude

**DOI:** 10.31486/toj.23.5038

**Published:** 2023

**Authors:** Ronald G. Amedee

**Affiliations:** Director of Clinical School and Professor, The University of Queensland Medical School, Ochsner Clinical School, New Orleans, LA; Editor-in-Chief, *Ochsner Journal*

*Gratitude makes sense of our past, brings peace for today, and creates a vision for tomorrow.*

*–Melody Beattie*

The Winter 2023 issue of the *Ochsner Journal* contains four original research articles, one innovative program report, two literature reviews, and seven case reports and clinical observations that serve as the principal elements for this edition. This issue also contains the fourth quarterly Health, Medicine, and Society column, this time authored by Ochsner staff physician Kevin Conrad.

Original research includes an article by Weinberger, Zeina, and Ashkenazi on “Misdiagnosis of Acute Appendicitis in the Emergency Department: Prevalence, Associated Factors, and Outcomes According to the Patients’ Disposition,” followed by “Short-Term Outcomes of Expedited Arthroscopic Tensionable Knotless Biologic Tuberoplasty for Massive Irreparable Rotator Cuff Tears” authored by Suri, Verma, Lim, et al. Next, please find work by Giraldo-Grueso, Bedi, Tafur-Soto, et al detailing “Zero to Hero? Reducing the Rate of Acute Kidney Injury in Transcatheter Aortic Valve Replacement: The Low Contrast Approach,” followed by “Trends in Cigarette Smoking Among United States Adolescents” authored by Mejia, Adele, Levine, et al.

Ochsner Clinical School physician education leaders Denton and Seoane offer the innovative program report, coauthored by The University of Queensland Medical School faculty member Eley, on “Ten Years of a Trans-Pacific Medical Education Partnership–Training Globally to Serve Locally.” Our literature reviews in this edition include a paper from Ott, Barber, and Jordan entitled “Trends in Patient-Reported Outcomes Measurement Information System (PROMIS) Utilization in Orthopedic Sports Medicine: A Scoping Review,” followed by “Efficacy and Safety of Psychedelics in Treating Anxiety Disorders” authored by Feulner, Sermchaiwong, Rodland, and Galarneau.

Case reports and clinical observations include “Late Airway Compromise Secondary to Dural Tear and Cerebrospinal Fluid Leak” authored by Hutson and McAllister, followed by “Thoracic Spinal Intradural Arachnoid Cyst With a Fulminant Course” authored by Ayantayo, Owagbemi, Rasskazoff, and former Ochsner neurologist Wale Sulaiman. Next is a submission from Jatoi, Ashraf, Shaikh, et al reporting on “Tuberculosis Presenting as Ruptured Liver Abscess and Inferior Vena Cava Thrombosis in a Pediatric Patient.” Scullen, Milburn, Mathkour, and Amenta detail a case of “Endovascular Mechanical Thrombectomy for Right Hemispheric Stroke Syndrome Due to Acute Left A1-A2 Junction Thromboembolic Occlusion,” and next is “Bulbar Onset Amyotrophic Lateral Sclerosis in an African American Older Adult” written by Kasindi and Carstarphen. Rounding out this section are “Malignant Pheochromocytoma With Intravascular Extension to the Heart” by Haider, Khan, Islam, et al and “Dexmedetomidine Used to Maintain Spontaneous Ventilation in a Patient With Anterior Mediastinal Mass and Superior Vena Cava Syndrome” authored by Olix, Carollo, and Ural.

As we come to the end of another calendar and publishing year, I must extend my gratitude to all involved in the production of this quarterly publication. First, I must thank our Managing Editor, Kathleen McFadden, for all of the countless hours of reading and editing as she single-handedly ensures the overall quality of each edition of the *Ochsner Journal.* I am also grateful to all of the authors who submit their scholarly product for consideration of publication in the *Journal* and to our reviewers who donate their time and expertise providing written evaluations of the submitted articles. Finally, I am grateful to all of our readers and Ochsner Health leadership for supporting/sponsoring this body of work.

Happy holidays to each of you. Please enjoy quality time with your family and friends, and together let us do it all again in 2024!

